# Abstracts from Foreign Journals

**Published:** 1899-03

**Authors:** J. Preston Hoskins

**Affiliations:** Princeton, N. J.


### ABSTRACTS FROM FOREIGN JOURNALS.

### GERMAN.

Under the Direction of J. Preston Hoskins, Ph.D.,
PRINCETON, N. J.

Castration of Mares by Schwendimann in Bern. The author has been in the habit of castrating untamed mares which have not proved themselves susceptible to the ordinary means of breaking. The operation must be performed on the subject standing, without the use of narcotics.

A few days before the operation the animal is put upon half rations, and for twenty-four to thirty-six hours before the same must not be fed at all. In this state of hunger the animal is tied with a strong halter. A long strap or rein is passed in coils over the neck, back, and rump. A broad bellyband running beneath the chest is fastened to the sides of the stall to prevent the animal from kneeling, while a stout pole immediately in front of the hocks is shoved through the sides of the stall beneath the body, to prevent the animal from throwing itself down. If the stall is narrow enough to prevent side-movements these preparations will be sufficient. It is not necessary to hobble the hind feet, for the back of the stall can be fastened with stout boards as high as the fetlock. Absolute security against movement is, however, necessary to the success of the operation, for most animals try to defend themselves, often in a most unexpected manner; often, too, during the preparations, whence it is advisable to leave this only to responsible persons, so that the mare may not be needlessly excited and fagged out. The operator should avoid the same, while thoroughly cleaning out the rectum and washing and disinfecting the anal and vaginal region. After the tail has been bandaged with a wet linen cloth, and the operator's arm, hands, and finger-nails thoroughly cleansed and disinfected, he can wash out the vagina with a disinfecting fluid, lukewarm. The best is a sublimate solution of 0.5 : 1000. In spite of the fact that this irritates and often produces violent struggling, Schwendimann prefers it to all others.

Now follows the most important part of the operation—the incision into the vaginal arch or wall. As a rule, the sheath is stretched on the introduction of the hand by the stream of air which follows; but if this does not take place this condition must be induced by spreading the hand close to the vestibule. The incision into the vaginal wall is made with the bistouri caché, 3 cm. above the orificium externum and 1 cm. sideways from the medial line. The thrust should be bold and strong (there is no risk), straight toward the front and inclined downward a little. The small opening, which is often immediately covered with the folds of the mucous membrane, and hence hard to find, should be located by the index-finger before the blade is withdrawn, and made larger. If it becomes evident that the peritoneum has not been reached with the instrument, extreme caution is necessary not to try to perforate this with the finger, for this cannot be done, as it will retreat further and further, and a sort of cul-de-sac will be formed, which is equivalent to a complete failure of the operation. Under these circumstances the bistoury must be introduced again and the incision completed, which is generally not difficult.

After the opening has been enlarged so that the whole hand can be introduced, the long écraseur used for this purpose is applied with the right hand, the left ovary is entangled with its chain, and slowly twisted. The other hand must see to it that none of the intestinal coils are entangled with it, and that the ovary does not fall finally into the abdominal cavity. The right ovary is sought with the left hand and removed as on the left side, only, beforehand, the left arm and hand must be washed and disinfected from the stains received in the first operation.

After the removal of the second ovary the operation is complete. It is not at all necessary and would probably be dangerous to again wash out the vagina. The animal must be carefully watched as long as signs of struggling continue. The colic, which lasts for about an hour, is without significance. With aseptic treatment the much-feared vulnerability of the peritoneum is not to be seriously considered.—*Thierärzliches Centralblatt*, June 10, 1898.

Carbol Odor in Meat after Carbolineum has been Taken. Official Veterinarian Angst, in Lauenburg, in his work on compulsory slaughter by the sanitary police, relates the following incident: On a farm a heifer fell sick at the time when the barn and stalls had been painted with carbolineum. The animal was killed and the meat put on sale, because it looked healthy and had no unnatural odor. When the meat was cooked, however, partly together with pork, a very strong carbol odor developed. The butcher had to take back all the meat and pay for the pork which had been thus spoiled. Not until then did it come to light that the animal had taken carbolineum from a pot which the painters supposed somebody had overturned as a joke. The *Allgeomeine Fleischzeitung* cites a similar case in Eberswalde. Boiled and roasted meat which smelled so strongly of carbol that it could not be eaten was brought back to the butchers. The animals from which the meat came had stood in the stalls of slaughter-houses which had been freshly disinfected with carbol.—*Idem.*

Operation for Constipation on the Intestines and Stomach of a Dog. A Leonberg dog which had suffered for months from constipation, and had not responded to treatment, was handed over in a very poor condition to Professor Venneholm. Immediately in front of the pubic bone, by the side of the prepuce, a hard, movable tumor about the size of one's fist could be discovered. After disinfecting the region of operation an incision 10 cm. long was made in the linea alba, yet the portion of the intestine which contained the foreign body could not be gotten out through this. The incision was sewed up and a second made by the side of the prepuce, diagonally toward the front and outside. The A. and V. epigastrica posterior were bound up and cut through. The greatly enlarged portion of the intestine, which had become hypertrophied in the wall, was drawn out and an incision about 8 cm. long made in the intestinal wall. A fecal ball as large as the fist and hard as a stone was taken out and the intestinal cavity washed out. Lembert's intestinal seam was made, serosa against serosa, without penetrating the mucous. The wound healed. The favorable outcome is remarkable, inasmuch as the animal was run down and a double laparotomy had to be performed.

A large dog swallowed the 17 cm. fibula of a horse. Violent and continued vomiting followed. On account of the danger of perforating the stomach emetics were avoided and an operation preferred. The animal was put under the influence of morphine and laparotomy performed in the linea alba on the navel. The incision was 2 cm. long, the bone was removed, and the wound sewed with a double thread of catgut. The animal recovered.—*Idem.*

---

Villate's solution, prepared from oxide of lead, Goulard's extract and vinegar are said to heal quittors in fifteen days. The solution is repeatedly injected into the fistulous tracts, and no other treatment is required. What a pity if this is not true?

